# Personality disorders, depression and anxiety in mothers of children with ADHD and anxiety disorders in Iran

**DOI:** 10.34763/jmotherandchild.20222601.d-22-00016

**Published:** 2022-11-02

**Authors:** Mohsen Dadashi, Roshanag Bateni, Abolfazl Ghoreishi

**Affiliations:** Social Determinants of Health Research Center, Department of Clinical Psychology, Zanjan University of Medical Sciences, Zanjan, Iran; Zanjan University of Medical Sciences, Zanjan, Iran; Social Determinant of Health Research Center, Metabolic Diseases Research Center, Department of Psychiatry, Zanjan University of Medical Sciences, Zanjan, Iran

**Keywords:** Psychopathology, personality, attention deficit hyperactivity disorder, anxiety, depression, parenting

## Abstract

**Background/aim:**

This study aims to assess and compare personality disorders and psychiatric disorders (depression and anxiety) in mothers of children with ADHD and anxiety disorders aged 2–16 years living in Iran.

**Material and methods:**

This is a descriptive cross-sectional study. Participants were 168 mothers (100 with children having ADHD and 68 with children having anxiety disorders). The Millon Clinical Multiaxial Inventory-III, the Depression Anxiety Stress Scale (DASS-21) and the Symptom Checklist-90–Revised (SCL-90-R) were used for assessing personality disorders, depression and anxiety in mothers. Collected data were analysed in SPSS software.

**Results:**

Of 168 mothers, only 100 completed the questionnaires completely (68 having children with ADHD and 32 with anxious children). Of 100 mothers, 61 had personality disorders, where 21 had children with anxiety disorders and 40 had children with ADHD. The most common personality disorder was depressive personality disorder (n = 27) followed by compulsive personality disorder (n = 15). No antisocial, borderline and paranoid personality disorders were observed in mothers. Based on DASS-21, 72 mothers had depression, and 84 had anxiety. Based on the SCL-90-R, 86 had depression, and 81 had anxiety. We found no statistically significant difference between the two groups of mothers in terms of personality disorders, depression and anxiety.

**Conclusion:**

Prevalence of depression, anxiety and personality disorders in mothers of children with anxiety disorders and ADHD in Iran is high, and there is no difference between them. It is recommended that psychiatric and psychological counseling be provided for these mothers.

## Introduction

Raising a child with Attention Deficit Hyperactivity Disorder (ADHD) or anxiety disorders is challenging because their symptoms are linked to dysfunctional behaviours, yielding high levels of friction for family life [1]. ADHD is one of the most common mental disorders in childhood with symptoms, including inattention (not being able to keep focus), hyperactivity (excess movement that is not fitting to the setting) and impulsivity (hasty acts that occur in the moment without thought), and it affects 8.4% of children and 2.5% of adults [2, 3] with an overall pooled estimate of 7.2% [4]. It is more common among boys than girls [5]. It can cause academic failure, social skills problems and strained parent-child relationships [6]. It impacts not only on the child but also on parents and siblings, causing disturbances to family and marital functioning [7]. Parents and relatives of ADHD children are at high risk for ADHD, comorbid psychiatric disorders, school failure, learning disability and impairments in intellectual functioning [8, 9, 10]. A study in Iran showed that the lifetime prevalence of depression in mothers and fathers having children with ADHD is 48.1% and 43%, respectively [11]. There are some studies focused on the characteristics of mothers of children with ADHD. For example, Pimentel et al. [12] and Babakhanian et al. [13] showed that the mothers of children with ADHD experience higher levels of parenting stress and report more behavioural problems in their children. Sfelinioti and Livaditis [14] in a review study, found that maternal depressive disorder and children’s ADHD influence each other through multiple psychosocial and biological factors. Van Batenburg-Eddes et al. [15] and Ayani et al. [16] also found a strong association between maternal anxiety and depressive symptoms with ADHD symptoms in children. Treatment of children with ADHD can have a favourable impact on their mothers’ depressive symptoms, which can consequently reduce negative parental attitudes, diminish the risk of behavioural disorders in these children, and exert a positive effect on their treatment [17].

Mothers with ADHD may also have personality characteristics that can be maladaptive. Personality characteristics have as important roles in coping, decision-making and other aspects of parenting practices. The studies on the personality of these mothers based on the NEO five dimensions have reported low levels of conscientiousness and agreeableness [1, 18], and higher levels of neuroticism [10, 19]. Positive personality traits (high levels of conscientiousness, extraversion, agreeableness and openness) can buffer the stress of rearing a child with ADHD, whereas negative personality traits (high neuroticism) can aggravate parenting stress [1]. Personality disorders of parents can predispose their children to other psychiatric disorders [20]. Identifying these disorders can help them better understand and predict future behaviours and problems in their children.

Depression and anxiety in both children and adults are frequently comorbid, suggesting that parental depression may be associated with child anxiety in addition to child depression. Evidence suggest that the association of parent and child depression may occur through child anxiety [21, 22]. In the study by Affrunti and Woodruff-Borden [23], maternal worry and depression significantly predicted lower levels of maternal-reported child anxiety and maternal anxiety predicted higher levels of maternal-reported child anxiety. Hudson and Rapee [24] found that mothers of anxious children were more negative during the interactions than mothers of non-clinical children. Identifying the status of depression and anxiety in mothers, in order to improve psychoeducational and other treatment interventions to favour family well-being and reduce parental distress, is potentially beneficial.

There is a scant research on personality and psychological status of Iranian mothers with ADHD. In a pilot study, Dadashzadeh et al. [25] evaluated the personality profile of parents of children with ADHD using the Millon Clinical Multiaxial Inventory-III (MCMI-III) and reported that the most common personality disorders were depressive (25.3%), histrionic (20%) and compulsive (17.1%) disorders. They reported that personality disorders are prevalent in parents of children with ADHD in Iran, and mothers suffer from personality disorders more than fathers. Abdi and Narimani [26] compared the personality traits in mothers of children with Autism Disorder, ADHD and normal children. Using the NEO Five-Factor Inventory, their results showed that the mothers of children with ADHD had higher levels of neuroticism. We found no study in Iran on the personality profile and psychological status of mothers with ADHD in comparison with those of mothers with children suffering from anxiety disorders. In this regard, the present study aims to investigate personality disorders and psychiatric disorders (depression and anxiety) in mothers of children with ADHD and anxiety disorders in Iran and evaluate whether there are significant differences between them. We hypothesize that: (a) mothers of children with ADHD have more personality disorders than mothers of children with anxiety disorders, and (b) mothers of children with ADHD have more depression and anxiety than mothers of children with anxiety disorders.

## Material and methods

### Participants

This is a descriptive cross-sectional study. Participants were 168 mothers (100 with children having ADHD and 68 with children having anxiety disorders) who were selected using a convenience sampling method from among those visited the psychiatric clinic of Shahid Beheshti Hospital in Zanjan, Iran, in 2018. The inclusion criteria were: Having a child aged 2–16 years, having a child with ADHD (diagnosed by a psychiatrist according to a Structured Clinical Interview for DSM-5 and the Intermediate Visual and Auditory Continuous Performance Test score) or anxiety disorders (diagnosed by a psychiatrist according to the DSM-5 criteria and based on the Beck Anxiety Inventory score), literacy to read and write, and willingness to participate in the study. Exclusion criteria were lack of cooperation, return of incomplete questionnaires and having any physical/mental disability. Prior to study, a written informed consent was obtained from them after explaining the study objectives and instruments to them and assuring them of the confidentiality of their information.

### Measures

Mothers were then assessed using a demographic form (surveying birth order, number, and gender of children as well as marital status, occupational status, and educational level of mothers), the Millon Clinical Multiaxial Inventory (MCMIIII), the Depression Anxiety Stress Scale (DASS-21) and the Symptom Checklist-90–Revised (SCL-90-R). The MCMI–III is a 175-item true-false self-report tool that assesses 10 clinical syndromes (Axis I) and 14 personality disorders (Axis II) based on the DSM-5. Personality disorders include schizoid, avoidant, depressive, dependent, histrionic, narcissistic, antisocial, sadistic, compulsive, negativistic, masochistic, schizotypal, borderline and paranoid. The clinical syndromes include anxiety, somatoform, bipolar, dysthymia, alcohol dependence, dysthymia, drug dependence, post-traumatic stress disorder, thought disorder, major depression and delusional disorder. In this study, we used the Persian version of this inventory to identify the personality disorders of mothers, which was validated by Sharifi et al. [27]. According to them, MCMI-III subscales have very good validity (0.58– 0.83) in the Iranian population. The DASS-21 is a valid and reliable self-report questionnaire that measures the emotional states of depression, anxiety and stress. It has 21 items, 7 for each three subscales. The items are rated on a 4-point Likert scale from 0 (did not apply to me at all) to 3 (applied to me very much). We used the Persian version of DASS-21 prepared by Sahebi et al. [28] who reported its good validity and reliability for the Iranian population. SCL-90-R includes 90 items (symptoms) assessing nine symptomatic dimensions: somatization, obsessive-compulsive disorder, interpersonal sensitivity, depression, anxiety, hostility, phobic anxiety, paranoid ideation, and psychoticism [29]. Each item is rated on a 5-point scale ranging from 0 (not at all) to 4 (extremely). We used the Persian version of this tool validated by Ardakani et al. [30], who reported its highly acceptable reliability for Iranian population.

### Statistical analysis

Of 168 questionnaires, only 100 returned complete. Therefore, the data of 100 mothers (68 having children with ADHD and 32 with anxious children) were included in the statistical analysis carried out in SPSS software. Data were described using descriptive statistics (frequency, percentage) and analysed using chi-square test. Kolmogorov-Smirnov test results showed that the data distribution was normal (p > 0.05), and Levene’s test showed the equality of variances (p > 0.05). The significance level was set at 0.05.

## Results

### Characteristics of participants

Most of participants were married (97% in the ADHD group; and 100% in the anxiety group) and housewives (63.23% in the ADHD group and 65.62% in the anxiety group) with academic degrees (38.23% in the ADHD group and 43.75% in the anxiety group). Moreover, the majority of them had two children (57.35% in the ADHD group and 40.62% in the anxiety group); most of these children were firstborn and male. For more information, see Table 1. Results of the chi-square test showed no significant difference between the two groups in terms of demographic factors (p > 0.05, Table 1).

**Table 1:** Demographic characteristics of the study participants

Characteristics		Anxiety group (n = 32) %	ADHD group (n = 68) %	P value*
	1^st^	19 (59.37)	37 (54.41)	
Birth order of children	2^nd^	8 (25)	27 (39.70)	0.262
	3^rd^	3 (9.37)	3 (4.41)	
	4^th^	2 (6.25)	1 (1.47)	
	1	10 (31.25)	17 (25)	
Number of children	2	13 (40.62)	39 (57.35)	0.316
	3	7 (21.87)	11 (16.17)	
	4	2 (6.25)	1 (1.47)	
Gender of children	Male	27 (84.37)	50 (73.53)	0.229
	Female	5 (15.62)	18 (26.47)	
Marital status of mothers	Single	0 (0)	2 (2.94)	0.327
	Married	32 (100)	66 (97.05)	
Occupational status of mothers	Housekeeper	21 (65.62)	43 (63.23)	0.816
	Employed	11 (34.37)	25 (33.82)	
	Lower than high school	10 (31.25)	22 (32.35)	
Educational level of mothers	High school diploma	8 (25)	20 (29.41)	0.850
	Academic	14 (43.75)	26 (38.23)	

*Chi-square test

### Personality disorders in mothers

Based on the answers to the personality disorders subscale of MCMI-III, 61 mothers had personality disorders, where 21 (65.62%) had children with anxiety disorders, and 40 (58.8%) had children with ADHD (Table 2). The most common personality disorder among mothers in overall was depressive personality disorder (n = 27) followed by compulsive (n = 15), dependent (n = 12) and histrionic (n = 12) personality disorders. None of them had antisocial, borderline or paranoid personality disorders. Table 2 presents the frequency of personality disorders and mean scores for each group. Of 27 cases with depressive personality disorder, 10 (31.25%) were the mothers of children with anxiety disorders (mean = 60.62 ± 21.57), and 17 (25%) were the mothers of children with ADHD (mean = 61.07 ± 21.63). Results of the chi-square test (Table 2) showed no significant difference between the mothers in terms of personality disorders (p > 0.05).

**Table 2:** Frequency of personality disorders and their mean scores in each group

Personality disorder	Answer	N(%)		Mean ± SD		Sig.*
		Anxiety group (n = 32)	ADHD group (n = 68)	Anxiety group (n = 32)	ADHD group (n = 68)	
Schizoid	True	0 (0)	1 (1.47)	38.71 ± 20.46	47.07 ± 19.35	0.491
	False	32 (100)	67 (98.53)			
Avoidant	True	1 (3.2)	3 (4.42)	44.43 ± 21.91	47.02 ± 18.81	0.759
	False	31 (96.8)	65 (95.58)			
Depressive	True	10 (31.25)	17 (25)	60.62 ± 21.57	61.07 ± 21.63	0.511
	False	22 (68.75)	51 (75)			
Dependent	True	5 (15.63)	7 (10.29)	40.15 ± 25.43	34.80 ± 23.38	0.444
	False	27 (84.37)	61 (89.71)			
Histrionic	True	5 (15.63)	7 (10.29)	49.81 ± 23.02	49.19 ± 24.31	0.444
	False	27 (84.37)	61 (89.71)			
	True	0 (0)	4 (5.88)	41.96 ± 21.76	43.95 ± 21.60	
Narcissistic	False	32 (100)	64 (94.12)			0.161
Sadistic	True	1 (3.2)	1 (1.47)	45.59 ± 18.72	41.17 ± 17.22	0.581
	False	31 (96.8)	67 (98.53)			
Compulsive	True	3 (9.38)	12 (17.65)	32.09 ± 23.96	35.75 ± 27.89	0.280
	False	29 (90.62)	56 (82.35)			
Negativistic	True	2 (6.25)	4 (5.88)	46.59 ± 19.96	45.79 ± 20.37	0.094
	False	30 (93.75)	64 (94.12)			
Masochistic	True	0 (0)	1 (1.47)	40.40 ± 20.95	42.10 ± 20.54	0.960
	False	312 (100)	67 (98.53)			
Schizotypal	True	0 (0)	1 (1.4)	47.65 ± 14.32	44.19 ± 17.68	0.491
	False	31 (100)	68 (98.6)			

Note = None of mothers had antisocial, borderline or paranoid personality disorders; SD = Standard deviation, *Chi-square test

### Prevalence of depression in mothers

Based on the answers to the depression subscale of DASS-21, 72 mothers had depression (14 mild, 17 moderate, 26 severe and 15 extremely severe) overall. Table 3 presents the frequency of depression and mean scores for each group. Of 72 cases with depression, 21 (65.62%) were related to the mothers of children with anxiety disorders (mean = 14.31 ± 9.79) and 51 (75%) were related to the mothers of children with ADHD (mean = 17.29 ± 9.48). Results of the chi-square test (Table 3) showed no significant difference between the mothers in depression dimension of DASS-21 (p > 0.05).

**Table 3:** Frequency of depression and anxiety and their mean scores in each group

Subscale	Status	N(%)		Mean ± SD		Sig.*
		Anxiety group (n = 32)	ADHD group (n = 68)	Anxiety group (n = 32)	ADHD group (n = 68)	
Depression – DASS-21	With	21 (65.62)	51 (75)	14.31 ± 9.79	17.29 ± 9.48	0.702
	Without	11 (34.38)	17 (25)			
Anxiety- SCL-90-R	With	27 (84.37)	57 (83.82)	12.87 ± 8.01	15.16 ± 7.75	0.571
	Without	5 (15.63)	11 (16.18)			
Depression – SCL-90-R	With	27 (84.375)	59 (86.75)	2.40 ± 0.75	2.61 ± 0.75	0.507
	Without	5 (15.625)	9 (13.25)			
	With	24 (75)	57 (83.82)			
Anxiety- DASS-21	Without	8 (25)	11 (16.18)	3.18 ± 1.63	3.70 ± 1.54	0.542

SD = Standard deviation, *Chi-square test

Based on the answers to the depression subscale of SCL-90-R, 86 mothers had depression; 27 (84.37%) were mothers of children with anxiety disorders (mean = 2.40 ± 0.75) and 59 (86.75%) were mothers of children with ADHD (mean = 2.61 ± 0.75). Results of the chi-square test (Table 3) also showed no significant difference between the mothers in depression dimension of SCL-90-R (p > 0.05).

### Prevalence of anxiety in mothers

Based on the answers to the anxiety subscale of SCL-90-R, 84 mothers had anxiety (19 mild, 60 moderate and 5 severe) overall. Table 3 presents the frequency of anxiety and mean scores for each group. Of 84 cases with anxiety, 27 (84.37%) were related to the mothers of children with anxiety disorders (mean = 12.87 ± 8.01), and 57 (83.82%) were related to the mothers of children with ADHD (mean = 15.16 ± 7.75). Results of the chi-square test (Table 3) showed no significant difference between the mothers in anxiety subscale of SCL-90-R (p > 0.05).

Based on the answers to the anxiety subscale of DASS-21, 81 mothers had anxiety, 24 (75%) were mothers of children with anxiety disorders (mean = 3.18 ± 1.63), and 57 (83.82%) were mothers of children with ADHD (mean = 3.70 ± 1.54). Results of the chi-square test (Table 3) also showed no significant difference between the mothers in anxiety subscale of DASS-21 (p > 0.05).

### Comorbidity of personality disorders and psychiatric disorders in mothers

Comorbidity of personality disorders, depression and anxiety were reported in 38 out of 100 mothers, in overall. Among mothers of children with ADHD, personality disorders (schizoid, avoidant, depressive, narcissistic, compulsive, dependent, negativistic, histrionic and sadistic) were comorbid with depression and anxiety in 25 out of 68 mothers (36.76%). Among mothers of children with anxiety disorders, personality disorders (masochistic, dependent, negativistic, depressive and histrionic) were comorbid with depression and anxiety in 13 out of 32 mothers (40.62%).

### Mixed personality disorders in mothers

Out of 100 mothers, 23 had mixed personality disorders, of whom 12 were mothers of children with ADHD (three with depressive-compulsive, four with depressive-dependent, two with depressive-negativistic, one with depressive-avoidant, one with avoidant-masochistic, and one with dependent-negativistic personality disorders), and 11 were mothers of children with anxiety disorders (three with histrionic-compulsive, two with depressive-dependent, and others each with depressive-avoidant, depressive-compulsive, depressive-negativistic, dependent-sadistic, depressive-dependent-histrionic and schizoid-depressive-dependent negativistic personality disorders).

## Discussion

The purpose of this study was to assess personality disorders, depression and anxiety in mothers of 100 children (68 children with ADHD and 32 children with anxiety disorders) in Iran. Using the MCMI-III tool, results showed that 61 mothers (out of 100) had personality disorders (21 with anxious children and 40 having children with ADHD), which is higher than the global rate (10–20%) [31]. The most common personality disorder was depressive personality disorder followed by compulsive personality disorder. No antisocial, borderline and paranoid personality disorders were observed in mothers. Moreover, 72 mothers (out of 100) had depression based on the DASS-21 (21 with anxious children and 51 having children with ADHD), and 86 mothers had depression based on the SCL-90-R (27 with anxious children and 59 having children with ADHD). Furthermore, 84 (out of 100) had anxiety based the DASS-21 score (27 with anxious children and 57 having children with ADHD), and 81 had anxiety based on the SCL-90-R (24 with anxious children and 57 having children with ADHD). These indicate that the prevalence of depression and anxiety are high in the mothers of children with anxiety disorders and ADHD compared to its rate in mothers of healthy children [32]. Hajebi et al. [33] reported the 12-month prevalence of anxiety disorders in Iranian women as 19.4%. Gharraee et al. [34] reported the prevalence of major depressive disorder in Iranian women as 4.8%, and Pakizeh [35] reported the prevalence of personality disorders in Iranian women as 46.1%.

However, we found no statistically significant difference between the two groups of mothers in depression, anxiety and personality disorders, which rejects the hypotheses of this study. In the case-control study by Margary et al. in Italy [36], parents of children with ADHD reported higher levels of ADHD symptoms, depression and depressive personality disorders than parents of healthy children, where mothers displayed greater presence of depression. This is consistent with our results. Our results are also in agreement with the findings of Dadashzadeh et al. [25] conducted in Iran. In the study by Steinhausen et al. in Switzerland [10], parents of children with ADHD were most abnormal on all dimensions of ADHD psychopathology and the SCL-90-R.

ADHD and anxiety in children can greatly affect the health status of their mothers resulting in psychological disorders, including anxiety and depression. Mothers’ anxiety and depression disrupts mental, emotional and supportive care and support for children in the family. A study showed that children with ADHD whose mothers were depressed were less positive in their parent–child interaction [37]. Genetics also has a role in this association [38]. Research shows that parents and siblings of someone with ADHD are more likely to have ADHD themselves [39]. Genetics polymorphisms are associated with adult ADHD and personality disorder [40, 41]. Polymorphisms such as DAT1, DRD4, DRD5, 5HTT, HTR1B and SNAP25 are more common in children with ADHD [42, 43]. The GTP-binding RAS-like 2 gene (DIRAS2), which regulates neurogenesis, as well as protein phosphatase 2, regulatory subunit B, gamma (PPP2R2C) gene located in the 4P16 region, channel-interacting protein 4 (KCNIP4) and SPOCK gene, is also associated with adult ADHD and personality disorders [44, 45, 46, 47].

There were some limitations and disadvantages in this study including lack of a control group, the use of self-report tools, low sample size, not assessing fathers of children, not controlling effect of confounding factors (e.g. socioeconomic status and education) and not examining the effect size of children’s anxiety and ADHD on mothers’ personality profile and psychopathology. In this regard, further studies are recommended on fathers of children with anxiety and ADHD using controls for comparison, larger sample size from different cities, and other assessment tools. Generalisation of the results to all mothers of children with anxiety and ADHD in Iran should also be done with caution. Due to the high frequency of depression and anxiety and personality disorders in mothers of children with anxiety disorders and ADHD, it is recommended that psychiatric and psychological counseling be provided for these mothers.

## Conclusion

Prevalence of depression, anxiety and personality disorders in mothers of children with anxiety disorders and ADHD in Iran is high, and there is no difference between them. The occurrence of ADHD symptoms, psychopathology and personality disorders in parents of these children highlights the importance to integrate the treatment programs for children with the screening and treatment for psychopathological symptoms of the parents.

**Key points**

- In Iran, personality disorders are present in 65.62% of mothers of children with anxiety disorders and 58.8% of those with ADHD children;
- In Iran, depression is present in 65.62% of mothers of children with anxiety disorders and 75% of mothers of children with ADHD;
- In Iran, anxiety is present in 84.37% of mothers of children with anxiety disorders and in 83.82% of mothers of children with ADHD;
- There is no statistically significant difference between the two groups of mothers in terms of personality disorders, depression and anxiety;
- In 36.76% of mothers of children with ADHD, personality disorders are comorbid with depression and anxiety;
- In 40.62% of mothers of children with anxiety disorders, personality disorders are comorbid with depression and anxiety.

## References

[1] Perez Algorta G, Kragh CA, Arnold LE, Molina BSG, Hinshaw SP, Swanson JM (2018). Maternal ADHD Symptoms, Personality, and Parenting Stress: Differences Between Mothers of Children With ADHD and Mothers of Comparison Children. J Atten Disord.

[2] Danielson ML, Bitsko RH, Ghandour RM, Holbrook JR, Kogan MD, Blumberg SJ (2018). Prevalence of Parent-Reported ADHD Diagnosis and Associated Treatment Among U.S. Children and Adolescents, 2016. J Clin Child Adolesc Psychol.

[3] Simon V, Czobor P, Bálint S, Mészáros A, Bitter I (2009). Prevalence and correlates of adult attention-deficit hyperactivity disorder: meta-analysis. Br J Psychiatry.

[4] Thomas R, Sanders S, Doust J, Beller E, Glasziou P (2015). Prevalence of attention-deficit/hyperactivity disorder: a systematic review and meta-analysis. Pediatrics.

[5] Skogli EW, Teicher MH, Andersen PN, Hovik KT, Øie M (2013). ADHD in girls and boys--gender differences in co-existing symptoms and executive function measures. BMC Psychiatry.

[6] Mautone JA, Lefler EK, Power TJ (2011). Promoting Family and School Success for Children With ADHD: Strengthening Relationships While Building Skills. Theory Pract.

[7] Harpin VA (2005). The effect of ADHD on the life of an individual, their family, and community from preschool to adult life. Arch Dis Child.

[8] Wilens TE, Spencer TJ (2010). Understanding attention-deficit/hyperactivity disorder from childhood to adulthood. Postgrad Med.

[9] Epstein JN, Conners CK, Erhardt D, Arnold LE, Hechtman L, Hinshaw SP (2000). Familial aggregation of ADHD characteristics. J Abnorm Child Psychol.

[10] Steinhausen HC, Göllner J, Brandeis D, Müller UC, Valko L, Drechsler R (2013). Psychopathology and personality in parents of children with ADHD. J Atten Disord.

[11] Ghanizadeh A, Mohammadi MR, Moini R (2008). Comorbidity of psychiatric disorders and parental psychiatric disorders in a sample of Iranian children with ADHD. J Atten Disord.

[12] Pimentel MJ, Vieira-Santos S, Santos V, Vale MC (2011). Mothers of children with attention deficit/hyperactivity disorder: relationship among parenting stress, parental practices and child behaviour. Atten Defic Hyperact Disord.

[13] Babakhanian M, Sayar S, Babakhanian M, Mohammadi G (2016). Iranian Children With ADHD and Mental Health of Their Mothers: The Role of Stress. Iran J Psychiatry Behav Sci.

[14] Sfelinioti S, Livaditis M (2017). Association of maternal depression with children’s attention deficit hyperactivity disorder. Psychiatriki.

[15] Van Batenburg-Eddes T, Brion MJ, Henrichs J, Jaddoe VW, Hofman A, Verhulst FC (2013). Parental depressive and anxiety symptoms during pregnancy and attention problems in children: a cross-cohort consistency study. J Child Psychol Psychiatry.

[16] Ayano G, Betts K, Tait R, Dachew BA, Alati R (2021). Maternal depressive and anxiety symptoms and the risk of attention deficit hyperactivity disorder symptoms in offspring aged 17: Findings from the Raine Study. J Affect Disord.

[17] Gokcen C, Coskun S, Kutuk MO (2018). Comparison of Depression and Burnout Levels of Mothers of Children with Attention-Deficit Hyperactivity Disorder Before and After Treatment. J Child Adolesc Psychopharmacol.

[18] Nigg JT, John OP, Blaskey LG, Huang-Pollock CL, Willcutt EG, Hinshaw SP (2002). Big five dimensions and ADHD symptoms: links between personality traits and clinical symptoms. J Pers Soc Psychol.

[19] Weinstein CS, Apfel RJ, Weinstein SR (1998). Description of mothers with ADHD with children with ADHD. Psychiatry.

[20] Staroselsky A, Fantus E, Sussman R, Sandor P, Koren G, Nulman I (2009). Both parental psychopathology and prenatal maternal alcohol dependency can predict the behavioral phenotype in children. Paediatr Drugs.

[21] Colletti CJ, Forehand R, Garai E, Rakow A, McKee L, Fear JM (2009). Parent Depression and Child Anxiety: An Overview of the Literature with Clinical Implications. Child Youth Care Forum.

[22] Ohannessian CM, Hesselbrock VM, Kramer J, Kuperman S, Bucholz KK, Schuckit MA (2005). The relationship between parental psychopathology and adolescent psychopathology: An examination of gender patterns. J Emot Behav Disord.

[23] Affrunti NW, Woodruff-Borden J (2015). The effect of maternal psychopathology on parent-child agreement of child anxiety symptoms: A hierarchical linear modeling approach. J Anxiety Disord.

[24] Hudson JL, Rapee RM (2001). Parent-child interactions and anxiety disorders: an observational study. Behav Res Ther.

[25] Dadashzadeh H, Amiri S, Atapour A, Abdi S, Asadian M (2014). Personality profile of parents of children with attention deficit hyperactivity disorder. ScientificWorldJournal.

[26] Abdi R, Narimani A (2018). Comparison of Personality Traits and Resiliency in Mothers of Children with Autism Disorder and Attention Deficit /Hyperactivity Disorder with Normal Children’s Mothers. Middle Eastern Journal of Disability Studies (MEJDS).

[27] Sharifi AA, Moulavi H, Namdari K (2008). The validity of MCMI-III (Millon,1994) scales. Knowl Res Appli Psychol.

[28] Sahebi A, Asghari M, Salari R (2005). Validation of depression anxiety and stress scale (DASS-21) for an Iranian population. Journal of Iranian Psychologists.

[29] Derogatis LR (1992). SCL-90-R: Administration, scoring of procedures manual-II for the R (revised) version and other instruments of the psychopathology rating scale series (2nd ed.).

[30] Ardakani A, Seghatoleslam T, Habil H, Jameei F, Rashid R, Zahirodin A (2016). Construct Validity of Symptom Checklist-90-Revised (SCL-90-R) and General Health Questionnaire-28 (GHQ-28) in Patients with Drug Addiction and Diabetes, and Normal Population. Iran J Public Health.

[31] Sadock VJ, Sadock BA (2007). Kaplan & Sadock’s Synopsis of Psychiatry (10th ed).

[32] Ferrari AJ, Somerville AJ, Baxter AJ, Norman R, Patten SB, Vos T (2013). Global variation in the prevalence and incidence of major depressive disorder: a systematic review of the epidemiological literature. Psychol Med.

[33] Hajebi A, Motevalian SA, Rahimi-Movaghar A, Sharifi V, Amin-Esmaeili M, Radgoodarzi R (2018). Major anxiety disorders in Iran: prevalence, sociodemographic correlates and service utilization. BMC Psychiatry.

[34] Gharraee B, Zahedi Tajrishi K, Sheybani F, Tahmasbi N, Mirzaei M, Farahani H (2019). Prevalence of major depressive disorder in the general population of Iran: A systematic review and meta-analysis. Med J Islam Repub Iran.

[35] Pakizeh A (2012). Comparison of personality disorders in socially disadvantaged women and normal peers in Bushehr province. Pers Individ Differ.

[36] Margari F, Craig F, Petruzzelli MG, Lamanna A, Matera E, Margari L (2013). Parents psychopathology of children with Attention Deficit Hyperactivity Disorder. Res Dev Disabil.

[37] Lee PC, Lin KC, Robson D, Yang HJ, Chen VC, Niew WI (2013). Parent-child interaction of mothers with depression and their children with ADHD. Res Dev Disabil.

[38] Sadock BA, Sadock VJ, Ruiz P (2015). Kaplan & Sadock’s Synopsis of Psychiatry: Behavioral Sciences/Clinical Psychiatry (11th ed).

[39] Starck M, Grünwald J, Schlarb AA (2016). Occurrence of ADHD in parents of ADHD children in a clinical sample. Neuropsychiatr Dis Treat.

[40] Kent L, Doerry U, Hardy E, Parmar R, Gingell K, Hawi Z (2002). Evidence that variation at the serotonin transporter gene influences susceptibility to attention deficit hyperactivity disorder (ADHD): analysis and pooled analysis. Mol Psychiatry.

[41] Retz W, Thome J, Blocher D, Baader M, Rösler M (2002). Association of attention deficit hyperactivity disorder-related psychopathology and personality traits with the serotonin transporter promoter region polymorphism. Neurosci Lett.

[42] Gizer IR, Ficks C, Waldman ID (2009). Candidate gene studies of ADHD: a meta-analytic review. Hum Genet.

[43] Martel MM, Nikolas M, Jernigan K, Friderici K, Nigg JT (2010). Personality mediation of genetic effects on Attention-Deficit/Hyperactivity Disorder. J Abnorm Child Psychol.

[44] Jacob C, Nguyen TT, Weißflog L, Herrmann M, Liedel S, Zamzow K (2012). PPP2R2C as a candidate gene of a temperament and character trait-based endophenotype of ADHD. Atten Defic Hyperact Disord.

[45] Reif A, Nguyen TT, Weissflog L, Jacob CP, Romanos M, Renner TJ (2011). DIRAS2 is associated with adult ADHD, related traits, and co-morbid disorders. Neuropsychopharmacology.

[46] Weber H, Scholz CJ, Jacob CP, Heupel J, Kittel-Schneider S, Erhardt A (2014). SPOCK3, a risk gene for adult ADHD and personality disorders. Eur Arch Psychiatry Clin Neurosci.

[47] Weißflog L, Scholz CJ, Jacob CP, Nguyen TT, Zamzow K, Groß-Lesch S (2013). KCNIP4 as a candidate gene for personality disorders and adult ADHD. Eur Neuropsychopharmacol.

